# Neighborhood food environment and mortality among older Japanese adults: results from the JAGES cohort study

**DOI:** 10.1186/s12966-018-0732-y

**Published:** 2018-10-19

**Authors:** Yukako Tani, Norimichi Suzuki, Takeo Fujiwara, Masamichi Hanazato, Naoki Kondo, Yasuhiro Miyaguni, Katsunori Kondo

**Affiliations:** 1 Department of Global Health Promotion, Tokyo Medical and Dental University, 1-5-45, Yushima, Bunkyo-ku, Tokyo, 113-8519 Japan; 2 Japan Society for the Promotion of Science, 5-3-1, Kojimachi, Chiyoda-ku, Tokyo, 102-0083 Japan; 3 Center for Preventive Medical Sciences, Chiba University, 1-33 Yayoicho, Inage-ku, Chiba-shi, Chiba, 263-8522 Japan; 4 Department of Health Education and Health Sociology, School of Public Health, The University of Tokyo, 7-3-1 Hongo, Bunkyo-ku, Tokyo, 113-0033 Japan; 5 Department of Gerontological Evaluation, Center for Gerontology and Social Science, National Center for Geriatrics and Gerontology, 7-430 Morikoka-cho, Obu-shi, Aichi 474-8511 Japan; 6 Department of Social Preventive Medical Sciences, Center for Preventive Medical Sciences, Chiba University, 1-8-1 Inohana, Chuo-ku, Chiba-shi, Chiba, 260-8672 Japan

**Keywords:** Food environment, Food store availability, Mortality, Older adults, Japan

## Abstract

**Background:**

Previous research has linked lower availability of food stores selling fruits and vegetables to unhealthy diet. However, the longitudinal association between the availability of healthy food stores and mortality is unknown. This study examined the association between neighborhood availability of food stores and mortality by driving status among older adults.

**Methods:**

This study drew upon a three-year follow up of participants in the Japan Gerontological Evaluation Study, a population-based cohort study of Japanese adults aged 65 years or older. Mortality from 2010 to 2013 was analyzed for 49,511 respondents. Neighborhood availability of food stores was defined as the number of food stores selling fruits and vegetables within a 500-m or 1-km radius of a person’s residence. Both subjective (participant-reported) and objective (geographic information system-based) measurements were used to assess this variable. Cox regression models were used to estimate hazard ratios (HR) for mortality.

**Results:**

A total of 2049 deaths occurred during the follow up. Lower subjective availability of food stores was significantly associated with increased mortality. Compared with participants reporting the highest availability, the age- and sex-adjusted HR for those reporting the lowest availability was 1.28 (95% CI: 1.04–1.58; *p* = 0.02). The association remained significant after adjustment for sociodemographic (education, income, cohabitation, marital status, and employment status) and environmental (driving status, use of public transportation, and study site) status (HR = 1.24, 95% CI: 1.01–1.53, *p* = 0.04). This association was stronger among non-car users, among whom the HR for those reporting the lowest availability of food stores was 1.61 (95% CI: 1.08–2.41, *p* = 0.02). In contrast, no significant association was seen between objective availability and mortality.

**Conclusions:**

Lower availability of healthy food stores measured subjectively, but not objectively, was associated with mortality, especially among non-car users. Considering the decline in mobility with age, living in a neighborhood with many options for procuring fruits and vegetables within walking distance may be important for healthy aging.

**Electronic supplementary material:**

The online version of this article (10.1186/s12966-018-0732-y) contains supplementary material, which is available to authorized users.

## Background

Population aging is a global trend. In Japan, the proportion of people aged ≥60 years is the highest in the world, exceeding 30% [1]. This context can help to elucidate the secret of longevity. Along with its aging population, Japan’s family structures have also changed. Multigenerational living arrangements are becoming less common, and the majority of older people lives alone or with their spouses [2]. Thus, older adults are faced with the task of obtaining food on their own.

Several food-related environmental factors influence the ability to obtain healthy food; these factors include availability (adequacy of a healthy food supply—e.g., the number of food stores that sell healthy food near people’s homes), accessibility (travel time and distance), type and size of food stores, variety of items, price, and quality of food items [3, 4]. Among these factors, availability and accessibility are crucial for older people, because mobility (e.g., walking, driving a car, and using public transport) declines with age [5]. This decline affects older people’s diets and health outcomes [5–7]. However, most previous studies on food availability have been cross-sectional and, with the exception of studies in the United Kingdom, conducted in areas of low population density and therefore in environments with a low density of food stores (e.g., the United States (US), Australia, New Zealand, and Canada) [4, 8]. Population density, which takes into account both population and land area, is approximately one to two orders of magnitude higher in Japan than in these previous studies’ sites, with a population density of 348 people/km^2^ in Japan, compared with 35 people/km^2^ in the US, 0.3 people/km^2^ in Australia, 17 people/km^2^ in New Zealand, and 4 people/km^2^ in Canada [9]. The impact of food availability on health in Japan and other high-density food store environments remains unclear.

Furthermore, few food availability studies have focused on older adults [7, 10–12]. One intervention study showed that a mobile catering van that carried fresh food to housing sites for low-income seniors increased vegetable intake among older adults [13]. Beyond the association with diet, several studies have reported that limited availability of healthy food increases the risks of obesity and diabetes [14–16]. However, to our knowledge, no study has investigated the impact of the availability of healthy food on mortality. Moreover, previous studies have failed to account for mobility status.

To adequately evaluate the association between the availability of food stores and mortality, it is important to account for differences in measurement—the objective or subjective measurement of the availability of food stores. Objective measurement uses geographic information systems (GIS) to assess the exact number of food stores within a given distance. Subjective (perceived) measurement uses questionnaires to assess an individuals’ perceptions of food store availability in their neighborhoods [17–19]. Although most previous studies used objective measurements of availability [17, 18], a systematic review evaluating the association of objective and subjective measures of food store environments with dietary outcomes showed more consistent significant relationships for subjective measures than for objective measures [4]. We thus assessed both objective and subjective measures of food store availability in the present study.

We examined the impact of subjective and objective food store availability on mortality risk by driving status, using data from the Japan Gerontological Evaluation Study (JAGES), which is a large-scale, population-based cohort study of older people in Japan [20, 21]. We considered driving status an important variable because it was hypothesized that the effect of food store availability on mortality differs by mobility status.

## Methods

### Study design and participants

JAGES is a prospective cohort study established in 2010 to evaluate the social determinants of healthy aging among non-disabled older adults in Japan. This research drew on JAGES data from 15 municipalities in five prefectures (Hokkaido, Aomori, Yamanashi, Aichi, and Nagasaki). The baseline survey was conducted from August 2010 to December 2011. Self-administered questionnaires were distributed by mail to 86,967 people aged ≥65 years and who were physically and cognitively independent, were not eligible for benefits from the public long-term care insurance program, and lived independently in the community. The survey was conducted using random sampling in eight large municipalities and was administered to all eligible residents in seven small municipalities. A total of 56,910 questionnaires were returned (65% response rate), and 98% of them have been linked to death records (*N* = 55,510). The present analyses were carried out using data for 49,511 participants. We excluded respondents with missing data for residential address (*N* = 465) or sex (*N* = 2), and those who answered “unknown” (*N* = 710) or provided no answer (*N* = 2475) on the question about the subjective availability of food stores. Additionally, to ensure that the analytical sample included only participants who were actually physically and cognitively independent, we also excluded those who reported limitations (*N* = 983) or provided no answer (*N* = 1364) on the questions about activities of daily living (defined as being unable to walk, take a bath, or use the toilet without assistance [22]—these respondents were included in the JAGES accidentally). JAGES participants were informed that their participation in the study was voluntary and that completing and returning the questionnaire by mail indicated their consent to participate. The study protocol was approved by the Ethics Committees in the Research of Human Subjects at Nihon Fukushi University (№ 10–05) and Chiba University Faculty of Medicine (№ 1777).

### Mortality outcome

We determined vital status during the follow-up period from 2010 to 2013 (mean: 2.9 years; range: 2.1–3.5 years) by linking the cohort participants to mortality records in the national long-term care insurance database. There were a total of 2049 deaths in the analytical sample (cumulative mortality = 2049/49,511; 4.1%). The present study examined all-cause mortality instead of cause-specific mortality because death certificate data were unavailable.

### Subjective availability of food stores

Self-reported questionnaires were used to assess the subjective availability of food stores by asking, “How many stores or facilities selling fresh fruits and vegetables are located within 1 kilometer of your home?” Responses were given on a four-point Likert scale as follows: “many,” “some,” “few,” or “none” [10]. These responses corresponded to highest, middle–high, middle–low, and lowest subjective food availability, respectively. The responses were used instead of specific numbers because participants’ feelings of sufficiency or insufficiency may vary by neighborhood environment and social context.

### Objective availability of food stores

The 500-meter (m) mesh data from the Ministry of Economy, Trade and Industry Commerce Establishment Survey of 2007 [23] were used to assess the objective availability of food stores. We defined “food stores” as stores providing fresh fruits and vegetables. These included department stores, general merchandise stores, specialized supermarkets, and daily commodities stores. Because the association of food store availability with fruit and vegetable consumption varies by buffer distance [24], we calculated the number of stores along a straight line within a 500-m and 1-kilometer (km) radius of the center of participants’ residential community blocks (*chocho-aza,* the smallest administrative unit in Japan, roughly comparable to a US census-block group [25]). Assuming all types of stores were equally distributed within the 500-m mesh, we calculated the number of stores along a straight line within a 500-m and 1-km radius of the participants’ residences in accordance with the proportional distribution area of the map area (Additional file 1: Figure S1). ArcGIS 10.1 software was used for all spatial calculations. For the analyses, participants were categorized into quartiles based on the number of food stores within a 500-m or 1-km radius of their residence.

### Driving status

Participants were asked whether they drive a car by themselves or travel in a family member’s car when they go out. Driving status was classified as “non-car user” (for participants who neither drive a car by themselves nor travel in a family member’s car) or “car user” (for participants who either drive a car by themselves or travel in a family member’s car).

### Covariates

Each municipality provided data on the participants’ age at recruitment (65–69, 70–74, 75–79, or ≥ 80 years) and sex. Sociodemographic status, use of public transportation, walking and going out, and nutritional and health status were assessed using a self-reported questionnaire. Sociodemographic status included education level (≤ 9, 10–12, or ≥ 13 years, or other), annual household income (< 2.00, 2.00–3.99, or ≥ 4.00 million yen), living situation (lives with others or lives alone), marital status (married, widowed, divorced, never married, or other), and employment status (working, retired, or never worked) [26]. Annual household income was adjusted for household size, dividing the income by the square root of the number of people in the household. Environmental status included use of public transportation (train and bus) and the prefecture of residence (Hokkaido, Aomori, Yamanashi, Aichi, or Nagasaki). To determine use of public transportation, participants were asked whether they use a train and/or bus when they go out. Variables representing walking and going out included walking time (≥ 30 min/day or <  30 min/day) and frequency of going out (≥ 2 times/week or ≤ 1 time/week). Nutritional status included body mass index (BMI) and frequency of fruit/vegetable intake (≥ 1 time/day or <  1 time/day) and meat/fish intake (≥ 1 time/day or <  1 time/day) over the past month [10, 27]. We used standard categories of BMI to characterize participants as obese (≥ 30.0 kg/m^2^), overweight (25.0–29.9 kg/m^2^), normal (18.5–24.9 kg/m^2^), or underweight (< 18.5 kg/m^2^) [28]. Although this categorization was not specifically developed for older adults, a Japanese cohort study reported that both BMI <  18.5 kg/m^2^ and ≥ 30 kg/m^2^ were associated with increased risks of mortality among older adults [29]. Variables representing health status included medical treatment of diseases/symptoms and depressive symptoms. With multiple responses allowed, the participants were asked whether they were currently under medical treatment for cancer, heart disease, stroke, diabetes mellitus, respiratory diseases, or other diseases (hypertension, obesity, hyperlipidemia, osteoporosis, joint disease/neuralgia, external injury/fracture, gastrointestinal diseases, liver diseases, psychiatric diseases, dysphagia, visual impairment, hearing impairment, elimination problems, sleep disorders, unidentified diseases, and other) [20]. The JAGES used the Japanese version of the Geriatric Depression Scale-15 (GDS) [30] to measure levels of depressive symptoms among older adults and classify them into two groups: non-depressed (< 5) or depressed (≥ 5) [26, 31]. The population density of inhabitable land in participants’ residential school districts was calculated using the 2010 census and land-utilization tertiary mesh data (as of 2010) from the Ministry of Land, Infrastructure, Transport and Tourism’s National Land Numerical Information, based on a 1:25,000 topographic map of Japan. These calculations excluded non-developed areas (e.g., rivers, lakes, forests, and wasteland) [32]. Covariates with missing data were categorized as “missing.”

### Statistical analysis

Cox proportional hazards models were estimated, yielding hazard ratios (HR) and 95% confidence intervals (CI) for all-cause mortality over the three-year follow-up period. The following sequence of models was constructed: Model 1 was adjusted for age and sex; Model 2 was additionally adjusted for sociodemographic status, with education, annual income, living situation, marital status, and employment status as potential confounders; Model 3 was further adjusted for environmental status, with driving status, public transportation (train and bus) use, and prefecture of residence as covariates, to examine whether the relationship between food store availability and mortality was independent of other environmental factors; Model 4 was also adjusted for walking and going out (walking time and frequency of going out) as potential mediating factors linking food store availability to mortality; and Model 5 was further adjusted for nutritional status (BMI, frequency of fruit/vegetable and meat/fish intake), health status (medical treatment for cancer, heart disease, stroke, diabetes mellitus, respiratory diseases, or other diseases), and depressive symptoms as potential mediating factors. The analysis was stratified by driving status because we found a significant interaction between driving status and food store availability (Additional file 1: Table S1). All analyses were conducted using Stata, Version 14. Participants with missing data on covariates were included in the analysis.

## Results

Table 1 presents the baseline characteristics of the participants. Of all of the participants, 16.0% rated their subjective availability of food stores as “highest” and 5.4% rated it as “lowest.” The objective measurement showed participants with a high subjective availability tended to live in neighborhoods with more food stores, compared with those with a low subjective availability. The mean number of food stores within a 500-m radius of residence calculated by GIS (objective measure) was 12.3 for those who rated their subjective availability as “highest” and 5.5 for those who rated it as “lowest” (Additional file 1: Table S2). The objective measurement showed mean numbers of food stores within a 500-m radius of residence as 27, 11, 4, and 0.8 for the fourth (highest), third, second, and first (lowest) quartiles, respectively. Of all car users, 16% drove themselves and traveled in a family member’s car, 52% drove themselves only, and 32% traveled in a family member’s car only (data not shown in table). Non-car users also tended not to use public transportation; the percentages of both train and bus users among this group were less than 10% (Table 1). Most non-car users were categorized into the fourth quartile of objective availability of food stores, whereas most of car users were categorized into the first or second quartiles of objective availability of food stores. This means that non-car users tended to live in neighborhoods with many food stores, while car users tended to live in neighborhoods with few food stores.

**Table 1:** Baseline characteristics of older Japanese adults by driving status (*n* = 49,511)

	Total		Driving status			
			Non-car users		Car users	
	n = 49,511		n = 19,835		*n* = 29,676	
	N	%	N	%	N	%
Age (in years)						
65–69	15,785	31.9	5068	25.6	10,717	36.1
70–74	14,512	29.3	5983	30.2	8529	28.7
75–79	10,813	21.8	4877	24.6	5936	20.0
≥ 80	8401	17.0	3907	19.7	4494	15.1
Sex						
Male	23,092	46.6	8341	42.1	14,751	49.7
Female	26,419	53.4	11,494	57.9	14,925	50.3
Food store availability						
Subjective (perceived number of food stores within 1-km of residence)						
Highest	7898	16.0	3359	16.9	4539	15.3
Middle–high	30,013	60.6	12,102	61.0	17,911	60.4
Middle–low	8935	18.0	3565	18.0	5370	18.1
Lowest	2665	5.4	809	4.1	1856	6.3
Objective (mean number of food stores within 500-m radius of residence)						
Quartile 4 (27)	12,375	25.0	8696	43.8	3679	12.4
Quartile 3 (11)	12,010	24.3	5535	27.9	6475	21.8
Quartile 2 (4)	12,685	25.6	3249	16.4	9436	31.8
Quartile 1 (0.8)	12,441	25.1	2355	11.9	10,086	34.0
Objective (mean number of food stores within 1-km radius of residence)						
Quartile 4 (91)	12,369	25.0	9624	48.5	2745	9.2
Quartile 3 (36)	12,370	25.0	5297	26.7	7073	23.8
Quartile 2 (16)	12,378	25.0	2617	13.2	9761	32.9
Quartile 1 (4)	12,394	25.0	2297	11.6	10,097	34.0
Sociodemographic status						
Education (in years)						
Low (≤ 9)	22,736	45.9	8311	41.9	14,425	48.6
Middle (10–12)	16,250	32.8	6477	32.7	9773	32.9
High (≥ 13)	8147	16.5	3794	19.1	4353	14.7
Other/missing	2378	4.8	1253	6.3	1125	3.8
Annual income (in millions of yen)						
Low (< 2.00)	20,101	40.6	7898	39.8	12,203	41.1
Middle (2.00–3.99)	15,636	31.6	5856	29.5	9780	33.0
High (≥ 4.00)	4496	9.1	1759	8.9	2737	9.2
Missing	9278	18.7	4322	21.8	4956	16.7
Living situation						
Lives with others	41,237	83.3	15,006	75.7	26,231	88.4
Lives alone	6810	13.8	4070	20.5	2740	9.2
Missing	1464	3.0	759	3.8	705	2.4
Marital status						
Married	34,309	69.3	12,157	61.3	22,152	74.6
Widowed	10,294	20.8	4862	24.5	5432	18.3
Divorced	1694	3.4	1031	5.2	663	2.2
Never married	963	1.9	677	3.4	286	1.0
Other/missing	2251	4.5	1108	5.6	1143	3.9
Employment status						
Working	10,208	20.6	3501	17.7	6707	22.6
Retired	27,388	55.3	11,116	56.0	16,272	54.8
Never worked	5469	11.0	2378	12.0	3091	10.4
Missing	6446	13.0	2840	14.3	3606	12.2
Environmental status						
Driving status						
Car user	29,676	59.9	0	0	29,676	100
Public transportation						
Train user	9470	19.1	1650	8.3	7820	26.4
Bus user	6605	13.3	1908	9.6	4697	15.8
Prefecture of residence						
Hokkaido	4253	8.6	792	4.0	3461	11.7
Aomori	3057	6.2	727	3.7	2330	7.9
Yamanashi	3033	6.1	426	2.1	2607	8.8
Aichi	36,041	72.8	17,059	86.0	18,982	64.0
Nagasaki	3127	6.3	831	4.2	2296	7.7
Walking and going out						
Walking time						
≥ 30 min/day	31,072	62.8	12,290	62.0	18,782	63.3
< 30 min/day	15,345	31.0	6026	30.4	9319	31.4
Missing	3094	6.2	1519	7.7	1575	5.3
Frequency of going out						
≥ 2 times/week	39,544	79.9	14,819	74.7	24,725	83.3
≤ 1 time/week	7039	14.2	2477	12.5	4562	15.4
Missing	2928	5.9	2539	12.8	389	1.3
Nutritional status						
Body mass index (kg/m^2^)						
Underweight (< 18.5)	3354	6.8	1582	8.0	1772	6.0
Normal (18.5–24.9)	32,836	66.3	13,065	65.9	19,771	66.6
Overweight (25.0–29.9)	9222	18.6	3319	16.7	5903	19.9
Obese (≥ 30.0)	1134	2.3	440	2.2	694	2.3
Missing	2965	6.0	1429	7.2	1536	5.2
Frequency of vegetable/fruit intake						
≥ 1 time/day	37,209	75.2	13,861	69.9	23,348	78.7
< 1 time/day	9451	19.1	3555	17.9	5896	19.9
Missing	2851	5.8	2419	12.2	432	1.5
Frequency of meat/fish intake						
≥ 1 time/day	19,282	38.9	7284	36.7	11,998	40.4
< 1 time/day	27,094	54.7	9980	50.3	17,114	57.7
Missing	3135	6.3	2571	13	564	1.9
Health status						
Undergoing medical treatment						
Cancer (yes)	2134	4.3	903	4.6	1231	4.1
Heart disease (yes)	5983	12.1	2390	12.0	3593	12.1
Stroke (yes)	669	1.4	251	1.3	418	1.4
Diabetes mellitus (yes)	6247	12.6	2448	12.3	3799	12.8
Respiratory disease (yes)	1795	3.6	673	3.4	1122	3.8
Other (yes)	34,210	69.1	13,878	70.0	20,332	68.5
Depressive symptoms						
Non-depressed (GDS < 5)	29,877	60.3	11,264	56.8	18,613	62.7
Depressed (GDS ≥ 5)	11,365	23.0	4656	23.5	6709	22.6
Missing	8269	16.7	3915	19.7	4354	14.7

GDS = Geriatric Depression Scale

Among all participants, the incidence rate of death per 100,000 person-years was 3.6 for participants with the highest subjective availability of food stores, compared with 4.4 for participants with the lowest subjective availability (Table 2). Regarding objective availability of food stores within a 500-m radius of residence, the incidence rate of death per 100,000 person-years was 3.7 for participants in the fourth (highest) quartile of food store availability, compared with 4.3 for participants in the first (lowest) quartile of availability. Table 3 shows the results of multivariate Cox regression models. After adjusting for age and sex, lower subjective availability was associated with mortality (HR = 1.21, 95% CI: 1.04–1.41 for middle–low subjective availability and HR = 1.28, 95% CI: 1.04–1.58 for lowest subjective availability; *p* for trend < 0.01) (Table 3, Model 1). After further adjusting for sociodemographic and environmental status, these HRs were attenuated but remained statistically significant (HR = 1.19, 95% CI: 1.02–1.39 for middle–low subjective availability and HR = 1.24, 95% CI: 1.01–1.53 for lowest subjective availability; *p* for trend = 0.01) (Model 3). After adjusting for potential mediators (walking and going out, nutritional and health status), these HRs diminished and became statistically non-significant (Models 4 and 5). In contrast, there was no significant association between the objective availability of food stores in a 500-m radius and mortality. When we conducted the analysis using a 1-km radius instead of a 500-m radius, we still observed no significant association for the objective measurement of food store availability (data not shown in table).

**Table 2:** All-cause mortality by driving status among older Japanese adults (*n* = 49,511) during the 3-year follow-up period

	Total (n = 49,511)		Driving status			
			Non-car users (*n* = 19,835)		Car users (*n* = 29,676)	
	Number of deaths (%)	Incidence rate per 100,000 person-years (95% CI)	Number of deaths (%)	Incidence rate per 100,000 person-years (95% CI)	Number of deaths (%)	Incidence rate per 100,000 person-years (95% CI)
Food store availability						
Subjective availability						
Highest	291 (3.7)	3.6 (3.2–4.0)	77 (2.3)	2.7 (2.1–3.4)	214 (4.7)	4.1 (3.6–4.7)
Middle–high	1235 (4.1)	3.9 (3.7–4.2)	424 (3.5)	4.0 (3.6–4.4)	811 (4.5)	3.9 (3.6–4.2)
Middle–low	396 (4.4)	4.2 (3.8–4.6)	144 (4.0)	4.5 (3.8–5.3)	252 (4.7)	4.1 (3.6–4.6)
Lowest	127 (4.8)	4.4 (3.7–5.2)	35 (4.3)	4.5 (3.3–6.3)	92 (5.0)	4.3 (3.5–5.3)
Objective availability within a 500-meter radius of residence						
Quartile 4 (highest)	410 (3.3)	3.7 (3.4–4.1)	234 (2.7)	3.4 (3.0–3.9)	176 (4.8)	4.2 (3.7–4.9)
Quartile 3	462 (3.8)	3.8 (3.4–4.1)	183 (3.3)	3.8 (3.3–4.4)	279 (4.3)	3.8 (3.3–4.2)
Quartile 2	564 (4.4)	3.9 (3.6–4.3)	133 (4.1)	4.1 (3.4–4.8)	431 (4.6)	3.9 (3.5–4.3)
Quartile 1 (lowest)	613 (4.9)	4.3 (4.0–4.7)	130 (5.5)	5.1 (4.3–6.1)	483 (4.8)	4.1 (3.8–4.5)
Objective availability within a 1-kilometer radius of residence						
Quartile 4 (highest)	360 (2.9)	3.4 (3.1–3.8)	244 (2.5)	3.3 (2.9–3.7)	116 (4.2)	3.8 (3.2–4.6)
Quartile 3	485 (3.9)	3.7 (3.4–4.1)	170 (3.2)	3.6 (3.1–4.2)	315 (4.5)	3.8 (3.4–4.3)
Quartile 2	559 (4.5)	4.0 (3.6–4.3)	135 (5.2)	4.8 (4.1–5.7)	424 (4.3)	3.7 (3.4–4.1)
Quartile 1 (lowest)	645 (5.2)	4.5 (4.2–4.9)	131 (5.7)	5.2 (4.4–6.2)	514 (5.1)	4.4 (4.0–4.8)

*CI* confidence interval

**Table 3:** Hazard ratios with 95% confidence intervals for the association of mortality with subjective and objective availability of food stores among older Japanese adults (*n* = 49,511)

	Model 1	Model 2	Model 3	Model 4	Model 5
	HR (95% CI)	HR (95% CI)	HR (95% CI)	HR (95% CI)	HR (95% CI)
Food store availability					
Subjective availability					
Highest	ref	ref	ref	ref	ref
Middle–high	1.12 (0.99–1.27)	1.11 (0.98–1.26)	1.12 (0.99–1.28)	1.09 (0.95–1.23)	1.09 (0.95–1.23)
Middle–low	1.21 (1.04–1.41)^*^	1.18 (1.01–1.37)^*^	1.19 (1.02–1.39)^*^	1.11 (0.95–1.29)	1.05 (0.90–1.23)
Lowest	1.28 (1.04–1.58)^*^	1.25 (1.01–1.54)^*^	1.24 (1.01–1.53)^*^	1.13 (0.91–1.39)	1.05 (0.85–1.30)
*p* for trend	<0.01	0.02	0.01	0.19	0.74
Objective availability within a 500-meter radius of residence					
Quartile 4 (highest)	ref	ref	ref	ref	ref
Quartile 3	0.99 (0.87–1.14)	0.98 (0.86–1.12)	0.98 (0.86–1.12)	0.96 (0.84–1.10)	0.95 (0.83–1.10)
Quartile 2	1.05 (0.93–1.20)	1.04 (0.91–1.18)	1.04 (0.91–1.19)	1.01 (0.88–1.15)	1.01 (0.89–1.16)
Quartile 1 (lowest)	1.10 (0.97–1.25)	1.07 (0.94–1.22)	1.05 (0.92–1.21)	1.00 (0.87–1.15)	1.01 (0.88–1.16)
*p* for trend	0.08	0.18	0.31	0.75	0.58

*HR* hazard ratio, *CI* confidence interval, ref reference group

Model 1: Adjusted for age and sex

Model 2: Model 1 + adjusted for sociodemographic status (education, annual income, living situation, marital status, and employment status)

Model 3: Model 2 + adjusted for environmental status (driving status, public transportation [train and bus], and prefecture of residence)

Model 4: Model 3 + adjusted for walking and going out (walking time and frequency of going out)

Model 5: Model 4 + adjusted for nutritional status (body mass index, frequency of fruit/vegetable and meat/fish intake) and health status (medical treatment for cancer, heart disease, stroke, diabetes mellitus, respiratory diseases, or other, and depressive symptoms)

*p < 0.05

The interaction between the subjective availability of food stores and driving status was significant, indicating that the effect of the subjective availability of food stores on mortality was attenuated when participants used cars (Additional file 1: Table S1). When stratified by driving status, the incidence rate of death per 100,000 person-years was 2.7 for participants with the highest subjective availability and 4.5 for those with the lowest subjective measurement among non-car users, compared with an incidence rates of death of 4.1 for participants with the highest subjective availability and 4.3 for participants with the lowest subjective availability among car users (Table 2). Table 4 demonstrates that lower subjective availability was associated with mortality among non-car users. Compared with the highest subjective availability of food stores, the HR adjusting for age and sex was 1.46 (95% CI: 1.15–1.86) for middle–high subjective availability, 1.64 (95% CI: 1.24–2.17) for middle–low subjective availability, and 1.61 (95% CI: 1.08–2.41) for lowest subjective availability (*p* for trend < 0.01) (Model 1). After further adjusting for sociodemographic and environmental status, the HRs were attenuated but remained statistically significant, except that for lowest subjective availability (HR = 1.41, 95% CI: 1.11–1.80 for middle–high; HR = 1.55, 95% CI: 1.17–2.05 for middle–low; and HR = 1.44, 95% CI: 0.96–2.16 for lowest subjective availability; *p* for trend = 0.01) (Model 3). This association remained significant even after adjusting for potential mediators (Models 4 and 5). In contrast to non-car users, no significant association between subjective availability and mortality was found among car users. Objective availability was not associated with mortality among those of either driving status.

**Table 4:** Hazard ratios with 95% confidence intervals for the association of mortality with subjective and objective availability of food stores by driving status among older Japanese adults

	Model 1	Model 2	Model 3	Model 4	Model 5
	HR (95% CI)	HR (95% CI)	HR (95% CI)	HR (95% CI)	HR (95% CI)
Driving status					
Non-car users (*n* = 19,835)					
Subjective availability					
Highest	ref	ref	ref	ref	ref
Middle–high	1.46 (1.15–1.86)^**^	1.44 (1.13–1.83)**	1.41 (1.11–1.80)**	1.36 (1.06–1.73)*	1.38 (1.08–1.76)*
Middle–low	1.64 (1.24–2.17)^***^	1.59 (1.20–2.10)**	1.55 (1.17–2.05)**	1.43 (1.08–1.89)*	1.37 (1.03–1.82)*
Lowest	1.61 (1.08–2.41)^*^	1.56 (1.04–2.33)*	1.44 (0.96–2.16)	1.28 (0.85–1.92)	1.21 (0.80–1.82)
*p* for trend	< 0.01	< 0.01	0.01	0.08	0.22
Objective availability within a 500-meter radius of residence					
Quartile 4 (highest)	ref	ref	ref	ref	ref
Quartile 3	1.06 (0.87–1.28)	1.04 (0.86–1.27)	1.00 (0.82–1.22)	0.98 (0.80–1.20)	0.98 (0.81–1.20)
Quartile 2	1.06 (0.85–1.32)	1.04 (0.84–1.30)	0.99 (0.79–1.24)	0.95 (0.76–1.19)	0.92 (0.73–1.15)
Quartile 1 (lowest)	1.19 (0.95–1.48)	1.15 (0.91–1.44)	0.99 (0.78–1.26)	0.92 (0.72–1.18)	0.97 (0.76–1.24)
*p* for trend	0.17	0.28	0.93	0.49	0.64
Car users (n = 29,676)					
Subjective availability					
Highest	ref	ref	ref	ref	ref
Middle–high	0.99 (0.86–1.16)	0.99 (0.85–1.15)	1.01 (0.86–1.17)	0.97 (0.84–1.13)	0.96 (0.83–1.12)
Middle–low	1.05 (0.87–1.26)	1.02 (0.85–1.23)	1.03 (0.86–1.24)	0.98 (0.81–1.17)	0.92 (0.76–1.11)
Lowest	1.14 (0.89–1.46)	1.12 (0.87–1.43)	1.13 (0.89–1.45)	1.04 (0.81–1.34)	0.96 (0.75–1.23)
*p* for trend	0.24	0.39	0.34	0.85	0.49
Objective availability within a 500-meter radius of residence					
Quartile 4 (highest)	ref			ref	ref
Quartile 3	0.89 (0.73–1.07)	0.87 (0.72–1.05)	0.87 (0.72–1.05)	0.85 (0.71–1.03)	0.85 (0.70–1.02)
Quartile 2	0.95 (0.80–1.14)	0.93 (0.78–1.11)	0.93 (0.78–1.11)	0.91 (0.77–1.09)	0.94 (0.79–1.13)
Quartile 1 (lowest)	0.98 (0.82–1.16)	0.95 (0.80–1.13)	0.95 (0.79–1.13)	0.92 (0.77–1.10)	0.93 (0.78–1.11)
*p* for trend	0.69	0.94	0.92	0.84	0.95

*HR* hazard ratio, *CI* confidence interval; ref = reference group

Model 1: Adjusted for age and sex

Model 2: Model 1 + adjusted for sociodemographic status (education, annual income, living situation, marital status, and employment status)

Model 3: Model 2 + adjusted for environmental status (public transportation (train and bus) and prefecture of residence)

Model 4: Model 3 + adjusted for walking and going out (walking time and frequency of going out)

Model 5: Model 4 + adjusted for nutritional status (body mass index, frequency of fruit/vegetable and meat/fish intake) and health status (medical treatment for cancer, heart disease, stroke, diabetes mellitus, respiratory diseases, or other, and depressive symptoms)

*p < 0.05; **p < 0.01; ***p < 0.001

## Discussion

To our knowledge, this was the first study to examine the association between the availability of healthy food stores and mortality using both subjective and objective availability measurements. Among all participants, we found that lower subjective, but not objective, availability of food stores was associated with mortality. This association persisted after adjusting for age, sex, sociodemographic characteristics, and environmental status; however, it became statistically non-significant after adjusting for walking and going out, nutritional status, and health status. When stratified by driving status, we found that lower subjective availability of food stores was associated with mortality among non-car users, but not car users. This association was attenuated, but remained significant, even after adjusting for walking and going out, nutritional status, and health status.

This study described the effect of subjective availability in the context of Japan’s considerably higher population density and higher density of food stores, compared with the previous study settings of the US, Australia, New Zealand, and Canada. In our study sites in Japan, a mean of 10.6 food stores were found to be within a 500-m radius of a participant’s residence, and the mean population density of inhabitable land [32] was 3674 people/km^2^. Our findings suggest that living in a neighborhood with many options for accessing fruits and vegetables, and having a perception of high food availability, may be important for healthy aging in Japan.

We found that subjective, not objective, availability was associated with mortality. This pattern was previously observed in a systematic review evaluating the relationship between the availability of food stores and diet: Subjective measurements were found to be more consistently related to healthy dietary outcomes than were objective measurements [4]. In addition, we found that the associations between subjective availability measurements and mortality were significant and largely similar after adjusting for population density (Additional file 1: Table S3), suggesting that this relationship was independent of population density. Regarding objective availability, because of differences in food store densities across the study sites (prefectures), participants from sites with high-store density were overrepresented in the highest quartile of the objective measure. Therefore, we also conducted the analyses separately by study site using site-specific categories (based on within-site distributions). This analysis yielded similar results, suggesting the non-significant relationship was independent of study site (data not shown in table).

There are three possible explanations for this pattern. First, subjective availability incorporated realized access to food stores in the local vicinity, whereas objective availability simply indicated potential access [33]. Food choice is influenced by both potential access (e.g., number, size, and location of food stores) and realized access (e.g., neighborhood of residence, vehicle access, public transportation, and financial resources) [3]. Although our subjective measure of availability asked respondents to indicate the number of stores within 1 km of their home, the perception of this 1-km radius could vary based on physical barriers in their area that are difficult to evaluate with GIS, including volume of street traffic or steep roads. We found that older adults who reported low subjective availability of food stores lived in hillier environments compared with those who reported high availability; the average land slope [32] was 4.5° in areas with the lowest food store availability and 2.5° in areas with the highest availability (Additional file 1: Table S4). For these reasons, the subjective measure may be more capable than the objective measure of accurately describing access to food stores. One way to account for realized access in the objective measure would be assessing road network buffers instead of circular buffers [18]. Further investigation using global positioning systems may elucidate the actual mechanism linking subjective, rather than objective, availability to mortality.

Second, the subjective measure of availability may capture a picture of relatively long-term food store availability. In contrast, the objective measure captures only a single time-point of food store availability. In Japan, the entry rate for food store businesses was around 15% and the exit rate was around 30% from 1997 to 2002 [34]. More than 90% of the participants had lived in the same municipality for ≥10 years, and the subjective measure might be more representative of a participant’s perception of long-term food store availability in their local area compared with the objective measurement of a single time-point. Future studies are needed to account for changes in the rates of the entry and exit of food store businesses, and for long-term effects.

Third, subjective measures may act as proxies for health consciousness. For example, older people with high levels of health consciousness may have detailed knowledge of food store availability in their neighborhood. If this is the case, raising health consciousness among older people is more important than increasing the number of food stores in setting with high density of food stores.

The impact of subjective availability was prominent among non-car users. This finding was plausible, as car users can shop outside their residential area. Because car use declines with age (less than half of older adults in Japan hold a driver’s license, with 43% of adults aged ≥65 years and 25% of adults aged ≥75 years holding a license in 2010 [35]) and may be abandoned after a change in cohabitation, such as after the death of a spouse or partner, living in a neighborhood with many options for accessing fresh fruits and vegetables within walking distance may reduce the risk of mortality among older adults.

The association between the subjective availability of food stores and mortality was attenuated after adjusting for walking time and frequency of going out, which suggests neighborhood availability of food stores may contribute to reducing mortality risk by preventing physical inactivity. After retirement, grocery shopping may be a main reason for going out. Older adults who live in areas with a high availability of food stores within walking distance may frequently go out on foot for grocery shopping. In contrast, those who live in areas with a low availability of food stores may use a car or go out less frequently. In our sample, we found participants with low subjective availability of food stores tended to have shorter walking times and went out less frequently than did those with high subjective availability (Additional file 1: Table S5).

Key strengths of this study were its assessment of the effects of both subjective and objective availability of food stores on mortality, accounting for driving status, exploring these relationships in a high-density setting, and using a large-scale, population-based cohort study of older Japanese people. Several limitations of this study should be noted. First, we did not account for the type and size of food stores or for the price of food items; potentially important factors for making healthy food choices [3]. However, it is difficult to confirm the possible extent and direction of the resulting bias. Second, we did not account for availability of unhealthy food stores, such as fast food. Nonetheless, more than 90% of Japanese older adults reported that they visit fast-food restaurants less than once a month [36]. Third, our analysis was limited to all-cause mortality. Future studies should examine cause-specific mortality, especially diet-related outcomes, to clarify the effects of food store availability in the neighborhood. Fourth, we calculated objective availability using secondary data sources, which have been found to underestimate and overestimate food access, compared with primary data sources collected through field observation [37]. However, because it is difficult to collect data through field observation in population-based studies, we used government data, which are more reliable than are other secondary data. Fifth, we were unable to collect subjective and objective data for the same time period, which may have caused a difference in results for these measures. Sixth, we lacked information on the person responsible for grocery shopping and the frequency of car use, which may explain some of the variance in the association between food store availability and mortality. For example, even if the participant reported low subjective availability, the person responsible for grocery shopping in their household might report subjective availability as high, or vice versa. Finally, although the participants were exclusively physically and cognitively independent older people, we cannot rule out the possibility of an inverse association. For example, a person with a weak physical condition might move to an area that is convenient for shopping. However, more than 80% of the participants in this study had lived in the same municipality for ≥30 years, and the results of the analyses limited to this subsample were generally similar to the results for the full sample (Additional file 1: Table S6). Additionally, the results were substantially similar when we excluded early deaths (within 1 year) from the analysis, (Additional file 1: Table S7).

## Conclusions

Lower availability of healthy food stores, measured subjectively, but not objectively, was associated with mortality among non-car users, but not car users. Considering the decline of mobility with age, living in a neighborhood with many options for accessing fruits and vegetables within walking distance may be important for healthy aging. Future studies should address the mechanisms underlying the differences in these measures of food store availability. To design age-friendly neighborhoods and to secure access to healthy food, it is important to further investigate the factors influencing the subjective availability of food stores among older populations.

## Additional file

Additional file 1: **Figure S1.** Methods of calculating objective availability of food stores. **Table S1**. Hazard ratios with 95% confidence intervals for the association of mortality with subjective and objective availability of food stores among older Japanese adults (*n* = 49,511). **Table S2**. Number of food stores calculated by geographic information systems by subjective availability of food stores among older Japanese adults. **Table S3**. Hazard ratios with 95% confidence intervals for the association of mortality with subjective availability of food stores among non-car users (*n* = 19,835). Table S4. Land slope in the school district of residence by subjective availability of food stores in older Japanese adults. **Table S5**. Baseline characteristics of older Japanese adults by subjective availability of food stores (n = 49,511). **Table S6**. Hazard ratios with 95% confidence intervals for the association of mortality with subjective and objective availability of food stores by driving status among older Japanese adults who had lived in the same municipality for ≥30 years. **Table S7**. Hazard ratios with 95% confidence intervals for the association of mortality with subjective and objective availability of food stores by driving status among older Japanese adults after excluding early deaths (within 1 year). (DOCX 190 kb)

## Abbreviations

95% CI: 95% confidence interval
BMI: Body mass index
GDS: Geriatric Depression Scale
GIS: Geographic information system
HR: Hazard ratio
JAGES: Japan Gerontological Evaluation Study
km: Kilometer
m: Meter
US: United States
