# Supplementary material for: Neighborhood food environment and mortality among older Japanese adults: results from the JAGES cohort study
Source: Int J Behav Nutr Phys Act. 2018 Oct 19;15:101. doi: 10.1186/s12966-018-0732-y (PMC6194719; doi:10.1186/s12966-018-0732-y)
Supplement: Supplementary file 1 — Figure S1. Methods of calculating objective availability of food stores. Table S1. Hazard ratios with 95% confidence intervals for the association of mortality with subjective and objective availability of food stores among older Japanese adults (n = 49,511). Table S2. Number of food stores calculated by geographic information systems by subjective availability of food stores among older Japanese adults. Table S3. Hazard ratios with 95% confidence intervals for the association of mortality with subjective availability of food stores among non-car users (n = 19,835). Table S4. Land slope in the school district of residence by subjective availability of food stores in older Japanese adults. Table S5. Baseline characteristics of older Japanese adults by subjective availability of food stores (n = 49,511). Table S6. Hazard ratios with 95% confidence intervals for the association of mortality with subjective and objective availability of food stores by driving status among older Japanese adults who had lived in the same municipality for ≥30 years. Table S7. Hazard ratios with 95% confidence intervals for the association of mortality with subjective and objective availability of food stores by driving status among older Japanese adults after excluding early deaths (within 1 year). (DOCX 190 kb) [file 12966_2018_732_MOESM1_ESM.docx]

**Figure S1.**　Methods of calculating objective availability of food stores


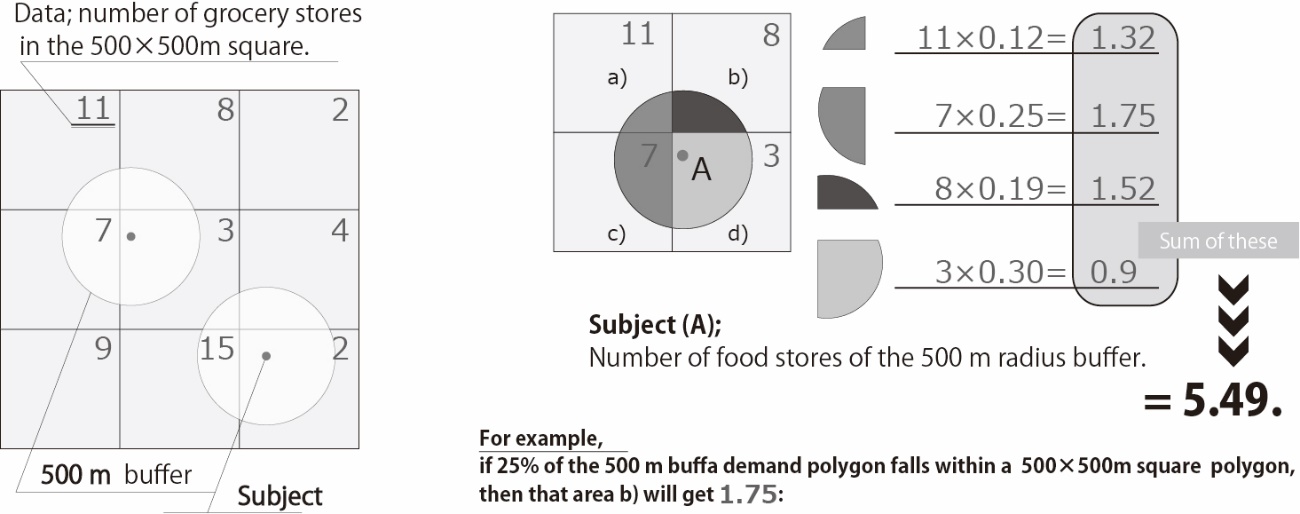


Assuming that all types of stores are equally distributed within the 500–meter (m) mesh, the number of stores along a straight line within a 500–m and 1–kilometer (km) radius of the participants’ residence was calculated in accordance with the proportional distribution area of the Geographic Information System (GIS)

**Table S1.** Hazard ratios with 95% confidence intervals for the association of mortality with subjective and objective availability of food stores among older Japanese adults (n = 49,511)

|  |  |  | HR (95% CI) | *p* |
| --- | --- | --- | --- | --- |
| Subjective availability | | | | |
|  | Subjective availability | |  |  |
|  |  | Highest | ref |  |
|  |  | Middle–high | 1.47 (1.15–1.87) | <0.01 |
|  |  | Middle–low | 1.65 (1.25–2.17) | <0.001 |
|  |  | Lowest | 1.62 (1.08–2.41) | 0.02 |
|  | Driving status | |  |  |
|  |  | Non-car user | ref |  |
|  |  | Car user | 1.43 (1.10–1.86) | <0.01 |
|  | Subjective availability x driving status | |  |  |
|  |  | Middle–high x Car user | 0.68 (0.51–0.90) | <0.01 |
|  |  | Middle–low x Car user | 0.64 (0.46–0.89) | <0.01 |
|  |  | Lowest x Car user | 0.71 (0.45–1.14) | 0.16 |
| Objective availability within a 500-meter radius of residence | | | | |
|  | Objective availability | |  |  |
|  |  | Quartile 4 (highest) | ref |  |
|  |  | Quartile 3 | 1.10 (0.90–1.33) | 0.35 |
|  |  | Quartile 2 | 1.15 (0.93–1.43) | 0.19 |
|  |  | Quartile 1 (lowest) | 1.30 (1.04–1.61) | 0.02 |
|  | Driving status | |  |  |
|  |  | Non-car user | ref |  |
|  |  | Car user | 1.19 (0.98–1.45) | 0.09 |
|  | Objective availability x driving status | |  |  |
|  |  | Quartile 3 x Car user | 0.81 (0.61–1.06) | 0.12 |
|  |  | Quartile 2 x Car user | 0.82 (0.62–1.08) | 0.16 |
|  |  | Quartile 1 x Car user | 0.75 (0.57–0.99) | 0.04 |

HR = hazard ratio; CI = confidence interval; ref = reference group

The models were adjusted for age and sex

**Table S2.** Number of food stores calculated by geographic information systems by subjective availability of food stores among older Japanese adults

|  |  | Number of food stores calculated by GIS (count)^a^ | | | | |
| --- | --- | --- | --- | --- | --- | --- |
|  |  | Food stores within a 500-meter radius of residence | |  | Food stores within a 1-kilometer radius of residence | |
|  |  | mean | SD |  | mean | SD |
| Subjective availability | | | | | | |
|  | Highest | 12.3 | 12.6 |  | 43.1 | 41.7 |
|  | Middle–high | 11.0 | 11.4 |  | 38.3 | 38.9 |
|  | Middle–low | 9.1 | 10.7 |  | 32.0 | 37.9 |
|  | Lowest | 5.5 | 9.0 |  | 19.8 | 30.0 |
|  | *p* for trend^b^ | < 0.001 |  |  | < 0.001 |  |

GIS = geographic information systems; SD = standard deviation

^a^The number of food stores along a straight line within a 500–meter or 1–kilometer radius of each participant’s residence was calculated in accordance with the proportional distribution area of the GIS (Supplementary Figure S1).

^b^*p* values were derived from Jonckheere–Terpstra trend tests across the subjective availability of food stores (highest, middle–high, middle–low, and lowest).

**Table S3.** Hazard ratios with 95% confidence intervals for the association of mortality with subjective availability of food stores among non-car users (n=19,835).

|  |  |  | Model 3 + population density^a^ | |  | Model 4 + population density^a^ | |  | Model 5 + population density^a^ | |
| --- | --- | --- | --- | --- | --- | --- | --- | --- | --- | --- |
|  |  |  | HR (95% CI) | *p* |  | HR (95% CI) | *p* |  | HR (95% CI) | *p* |
| Subjective availability | | |  |  |  |  |  |  |  |  |
|  |  | Highest | ref |  |  | ref |  |  | ref |  |
|  |  | Middle–high | 1.39 (1.09–1.77) | <0.01 |  | 1.34 (1.05–1.71) | <0.01 |  | 1.36 (1.07–1.74) | 0.01 |
|  |  | Middle–low | 1.50 (1.14–1.99) | <0.01 |  | 1.40 (1.05–1.85) | 0.02 |  | 1.34 (1.01–1.78) | 0.04 |
|  |  | Lowest | 1.38 (0.92–2.07) | 0.12 |  | 1.24 (0.83–1.87) | 0.29 |  | 1.18 (0.78–1.78) | 0.43 |
|  |  | *p* for trend | 0.03 |  |  | 0.12 |  |  | 0.29 |  |

HR = hazard ratio; CI = confidence interval; ref = reference group

^a^Adjusted for quartile of population density of inhabitable area in participants’ residential school districts

Model 3: Adjusted for age, sex, sociodemographic (education, annual income, living situation, marital status, and employment status) and environmental (public transportation [train and bus] and prefecture of residence) status

Model 4: Model 3 + adjusted for walking and going out (walking time and frequency of going out)

Model 5: Model 4 + adjusted for nutritional status (body mass index, frequency of fruit/vegetable and meat/fish intake) and health status (medical treatment for cancer, heart disease, stroke, diabetes mellitus, respiratory diseases, or other, and depressive symptoms)

**Table S4.** Land slope in the school district of residence by subjective availability of food stores in older Japanese adults.

|  |  | Land slop (°)^a^ | | | | |
| --- | --- | --- | --- | --- | --- | --- |
|  |  | Total |  | Driving status | | |
|  |  |  |  | Non-car users |  | Car users |
|  |  | Mean (SD) |  | Mean (SD) |  | Mean (SD) |
| Subjective availability | |  |  |  |  |  |
|  | Highest | 2.5 (3.0) |  | 1.7 (2.2) |  | 3.0 (3.3) |
|  | Middle–high | 2.8 (3.0) |  | 2.0 (2.5) |  | 3.4 (3.2) |
|  | Middle–low | 3.4 (3.4) |  | 2.4 (3.0) |  | 4.1 (3.5) |
|  | Lowest | 4.5 (4.0) |  | 3.7 (4.1) |  | 4.9 (3.9) |

^a^The average land slope in the residential school districts was calculated using the Elevation, Degree of Slope 5th Mesh Data (as of 2011) of the National Land Numerical Information from the Ministry of Land, Infrastructure, Transport and Tourism in Japan, based on the Digital Map 50 m Grid (Elevation) from the Geospatial Information Authority of Japan (GSI) (Ministry of Land, Infrastructure, Transport and Tourism, 2016. In: National Land Numerical Information Elevation, Degree of Slope 5th Mesh Data. Ministry of Land, Infrastructure, Transport and Tourism, Tokyo).

**Table S5.** Baseline characteristics of older Japanese adults by subjective availability of food stores (n = 49,511)

|  |  |  | Subjective availability of food stores | | | | | | |
| --- | --- | --- | --- | --- | --- | --- | --- | --- | --- |
|  |  |  | Highest |  | Middle–high |  | Middle–low |  | Lowest |
|  |  |  | n = 7,898 |  | n = 30,013 |  | n = 8,935 |  | n = 2,665 |
|  |  |  | % |  | % |  | % |  | % |
| Walking and going out | | |  |  |  |  |  |  |  |
|  | Walking time | |  |  |  |  |  |  |  |
|  |  | ≥ 30 minutes/day | 66.1 |  | 63.0 |  | 60.6 |  | 57.6 |
|  |  | < 30 minutes/day | 27.4 |  | 31.0 |  | 33.1 |  | 34.6 |
|  |  | Missing | 6.5 |  | 6.0 |  | 6.3 |  | 7.8 |
|  | Frequency of going out | |  |  |  |  |  |  |  |
|  |  | ≥ 2 times/week | 84.0 |  | 80.9 |  | 75.8 |  | 70.3 |
|  |  | ≤ 1 time/week | 10.2 |  | 13.3 |  | 17.9 |  | 23.6 |
|  |  | Missing | 5.8 |  | 5.8 |  | 6.3 |  | 6.1 |
| Nutritional status | | |  |  |  |  |  |  |  |
|  | Body mass index (kg/m^2^) | |  |  |  |  |  |  |  |
|  |  | Underweight (< 18.5) | 6.4 |  | 6.8 |  | 6.9 |  | 7.3 |
|  |  | Normal (18.5–24.9) | 66.2 |  | 66.4 |  | 65.9 |  | 66.8 |
|  |  | Overweight (25.0–29.9) | 19.6 |  | 18.6 |  | 17.9 |  | 18.0 |
|  |  | Obesity (≥ 30.0) | 2.2 |  | 2.2 |  | 2.6 |  | 2.0 |
|  |  | Missing | 5.6 |  | 5.9 |  | 6.6 |  | 5.8 |
|  | Frequency of vegetable/fruit intake | |  |  |  |  |  |  |  |
|  |  | ≥ 1 time/day | 78.4 |  | 75.5 |  | 71.9 |  | 72.5 |
|  |  | < 1 time/day | 15.9 |  | 18.9 |  | 21.9 |  | 21.6 |
|  |  | Missing | 5.6 |  | 5.6 |  | 6.3 |  | 5.9 |
|  | Frequency of meat/fish intake | |  |  |  |  |  |  |  |
|  |  | ≥ 1 time/day | 42.6 |  | 38.9 |  | 36.4 |  | 37.6 |
|  |  | < 1 time/day | 51.4 |  | 54.8 |  | 56.9 |  | 55.9 |
|  |  | Missing | 6.0 |  | 6.3 |  | 6.7 |  | 6.5 |
| Health status | | |  |  |  |  |  |  |  |
|  | Undergoing medical treatment | |  |  |  |  |  |  |  |
|  |  | Cancer (yes) | 4.3 |  | 4.2 |  | 4.5 |  | 4.4 |
|  |  | Heart disease (yes) | 11.5 |  | 12.0 |  | 12.3 |  | 14.1 |
|  |  | Stroke (yes) | 1.3 |  | 1.4 |  | 1.3 |  | 1.7 |
|  |  | Diabetes mellitus (yes) | 12.5 |  | 12.7 |  | 12.5 |  | 11.7 |
|  |  | Respiratory disease (yes) | 3.3 |  | 3.5 |  | 4.2 |  | 4.5 |
|  |  | Other (yes) | 66.5 |  | 68.8 |  | 71.0 |  | 73.8 |
|  | Depressive symptoms | |  |  |  |  |  |  |  |
|  |  | Non-depressed (GDS < 5) | 68.3 |  | 61.2 |  | 53.5 |  | 50.3 |
|  |  | Depressed (GDS ≥ 5) | 16.1 |  | 22.2 |  | 29.2 |  | 30.9 |
|  |  | Missing | 15.6 |  | 16.6 |  | 17.3 |  | 18.8 |

GDS = geriatric depression scale

**Table S6.** Hazard ratios with 95% confidence intervals for the association of mortality with subjective and objective availability of food stores by driving status among older Japanese adults who had lived in the same municipality for ≥ 30 years

|  |  |  |  | Model 1 |  | Model 2 |  | Model 3 |  |
| --- | --- | --- | --- | --- | --- | --- | --- | --- | --- |
|  |  |  |  | HR (95% CI) |  | HR (95% CI) |  | HR (95% CI) |  |
| Driving status | | | |  |  |  |  |  |  |
|  | Non-car users (n = 16,443) | | |  |  |  |  |  |  |
|  |  | Subjective availability | |  |  |  |  |  |  |
|  |  |  | Highest | ref |  | ref |  | ref |  |
|  |  |  | Middle–high | 1.42 (1.08–1.86) | ^*^ | 1.40 (1.07–1.83) | ^*^ | 1.36 (1.04–1.79) | ^*^ |
|  |  |  | Middle–low | 1.57 (1.15–2.14) | ^**^ | 1.52 (1.11–2.08) | ^**^ | 1.46 (1.07–2.00) | ^*^ |
|  |  |  | Lowest | 1.84 (1.20–2.83) | ^**^ | 1.80 (1.17–2.76) | ^**^ | 1.62 (1.05–2.50) | ^*^ |
|  |  |  | *p* for trend | <0.01 |  | <0.01 |  | 0.02 |  |
|  |  | Objective availability within a 500–meter radius of residence | | | | |  |  |  |
|  |  |  | Quartile 4 (highest) | ref |  |  |  |  |  |
|  |  |  | Quartile 3 | 1.10 (0.88–1.36) |  | 1.09 (0.88–1.36) |  | 1.05 (0.85–1.31) |  |
|  |  |  | Quartile 2 | 1.04 (0.80–1.34) |  | 1.03 (0.80–1.33) |  | 1.00 (0.77–1.30) |  |
|  |  |  | Quartile 1 (lowest) | 1.26 (0.98–1.63) |  | 1.24 (0.96–1.61) |  | 1.11 (0.84–1.47) |  |
|  |  |  | *p* for trend | 0.13 |  | 0.17 |  | 0.57 |  |
|  | Car users (n = 23,898) | | |  |  |  |  |  |  |
|  |  | Subjective availability | |  |  |  |  |  |  |
|  |  |  | Highest | ref |  | ref |  | ref |  |
|  |  |  | Middle–high | 1.01 (0.86–1.20) |  | 1.01 (0.85–1.20) |  | 1.02 (0.86–1.22) |  |
|  |  |  | Middle–low | 1.09 (0.89–1.34) |  | 1.06 (0.86–1.30) |  | 1.07 (0.87–1.31) |  |
|  |  |  | Lowest | 1.19 (0.91–1.55) |  | 1.16 (0.88–1.51) |  | 1.18 (0.90–1.54) |  |
|  |  |  | *p* for trend | 0.15 |  | 0.26 |  | 0.22 |  |
|  |  | Objective availability within a 500–meter radius of residence | | | | |  |  |  |
|  |  |  | Quartile 4 (highest) | ref |  |  |  |  |  |
|  |  |  | Quartile 3 | 0.96 (0.78–1.18) |  | 0.94 (0.76–1.16) |  | 0.94 (0.76–1.16) |  |
|  |  |  | Quartile 2 | 1.00 (0.82–1.22) |  | 0.98 (0.80–1.19) |  | 0.98 (0.80–1.19) |  |
|  |  |  | Quartile 1 (lowest) | 1.09 (0.90–1.32) |  | 1.05 (0.87–1.28) |  | 1.06 (0.87–1.29) |  |
|  |  |  | *p* for trend | 0.19 |  | 0.32 |  | 0.30 |  |

HR = hazard ratio; CI = confidence interval; ref = reference group

Model 1: Adjusted for age and sex

Model 2: Model 1 + adjusted for sociodemographic status (education, annual income, living situation, marital status, and employment status)

Model 3: Model 2 + adjusted for environmental status (public transportation [train and bus], and prefecture of residence)

**p* < 0.05; ***p* < 0.01

**Table S7.** Hazard ratios with 95% confidence intervals for the association of mortality with subjective and objective availability of food stores by driving status among older Japanese adults after excluding early deaths (within 1 year)

|  |  |  |  | Model 1 |  | Model 2 |  | Model 3 |  |
| --- | --- | --- | --- | --- | --- | --- | --- | --- | --- |
|  |  |  |  | HR (95% CI) |  | HR (95% CI) |  | HR (95% CI) |  |
| Driving status | | | |  |  |  |  |  |  |
|  | Non-car users (n=19,568) | | |  |  |  |  |  |  |
|  |  | Subjective availability | |  |  |  |  |  |  |
|  |  |  | Highest | ref |  | ref |  | ref |  |
|  |  |  | Middle–high | 1.42 (1.06–1.90) | ^*^ | 1.39 (1.04–1.86) | ^*^ | 1.36 (1.02–1.82) | ^*^ |
|  |  |  | Middle–low | 1.55 (1.11–2.16) | ^*^ | 1.49 (1.07–2.08) | ^*^ | 1.46 (1.04–2.03) | ^*^ |
|  |  |  | Lowest | 1.34 (0.81–2.20) |  | 1.28 (0.78–2.11) |  | 1.19 (0.72–1.97) |  |
|  |  |  | *p* for trend | <0.01 |  | 0.10 |  | 0.17 |  |
|  |  | Objective availability within a 500–meter radius of residence | | | | |  |  |  |
|  |  |  | Quartile 4 (highest) | ref |  |  |  |  |  |
|  |  |  | Quartile 3 | 1.10 (0.86–1.39) |  | 1.09 (0.85–1.38) |  | 1.05 (0.82–1.34) |  |
|  |  |  | Quartile 2 | 1.01 (0.78–1.33) |  | 1.00 (0.77–1.31) |  | 0.97 (0.74–1.27) |  |
|  |  |  | Quartile 1 (lowest) | 1.18 (0.90–1.54) |  | 1.13 (0.86–1.48) |  | 1.01 (0.76–1.35) |  |
|  |  |  | *p* for trend | 0.34 |  | 0.51 |  | 0.94 |  |
|  | Car users (n=29,319) | | |  |  |  |  |  |  |
|  |  | Subjective availability | |  |  |  |  |  |  |
|  |  |  | Highest | ref |  | ref |  | ref |  |
|  |  |  | Middle–high | 0.93 (0.78–1.10) |  | 0.92 (0.78–1.09) |  | 0.93 (0.79–1.10) |  |
|  |  |  | Middle–low | 0.99 (0.81–1.22) |  | 0.97 (0.79–1.19) |  | 0.97 (0.79–1.19) |  |
|  |  |  | Lowest | 1.11 (0.85–1.46) |  | 1.09 (0.83–1.44) |  | 1.10 (0.84–1.45) |  |
|  |  |  | *p* for trend | 0.42 |  | 0.57 |  | 0.58 |  |
|  |  | Objective availability within a 500–meter radius of residence | | | | |  |  |  |
|  |  |  | Quartile 4 (highest) | ref |  |  |  |  |  |
|  |  |  | Quartile 3 | 0.88 (0.71–1.09) |  | 0.87 (0.70–1.07) |  | 0.87 (0.70–1.08) |  |
|  |  |  | Quartile 2 | 0.97 (0.80–1.18) |  | 0.95 (0.78–1.16) |  | 0.95 (0.78–1.16) |  |
|  |  |  | Quartile 1 (lowest) | 0.94 (0.77–1.14) |  | 0.91 (0.75–1.11) |  | 0.93 (0.76–1.13) |  |
|  |  |  | *p* for trend | 0.96 |  | 0.72 |  | 0.88 |  |

HR = hazard ratio; CI = confidence interval; ref = reference group

Model 1: Adjusted for age and sex

Model 2: Model 1 + adjusted for sociodemographic status (education, annual income, living situation, marital status, and employment status)

Model 3: Model 2 + adjusted for environmental status (public transportation [train and bus] and prefecture of residence)

**p* < 0.05
